# Exosome-transmitted miR-30a-5p enhances cell proliferation, migration, and invasion in ovarian cancer

**DOI:** 10.1186/s13008-023-00099-2

**Published:** 2023-11-01

**Authors:** Jian Cao, Huan Wang, Jing Yang, Jing Zhang, Dake Li, Pengfei Xu

**Affiliations:** 1 Department of Gynecology, Women’s Hospital of Nanjing Medical University (Nanjing Maternity and Child Health Care Hospital), 123 Tianfei Lane, Mochou Road, Qinhuai District, Nanjing, 210004 Jiangsu China; 2 Department of Gynecology, The First Affiliated Hospital of Nanjing Medical University, Nanjing, 210004 Jiangsu China; 3 Department of Gynecologic Oncology, The First Affiliated Hospital of Bengbu Medical College, Bengbu, 233004 Anhui China; 4 Nanjing Maternal and Child Health Institute, Women’s Hospital of Nanjing Medical University (Nanjing Maternity and Child Health Care Hospital), 123 Tianfei Lane, Mochou Road, Qinhuai District, Nanjing, 210004 Jiangsu China

**Keywords:** Ovarian cancer, Exosome, miR-30a-5p, Proliferation, Invasion, Migration

## Abstract

**Background:**

Ovarian cancer (OC) causes the highest rates of mortality among women’s genital tract malignancies. Micro-ribonucleic acid (miRNA), the most abundant long noncoding RNAs transmitted by exosomes, has been revealed to be a potential marker for OC since 2008. In this study, we aimed to determine the possible roles of miRNAs derived from exosomes in the early diagnosis of OC through miRNA microarray, besides, exploring the underlying mechanisms of miRNAs in the OC progression.

**Methods:**

We isolated exosomes from high invasive OC cell line HO8910PM and its parent cell line HO8910 using transmission electron microscopy and western blot, and performed miRNA microarray to identify the exosome-transmitted miRNA from the two cell lines, respectively. The expression profile was obtained by quantitative analysis, and then the differentially expressed individuals were screened. miRNA-30a-5p, a stable miRNA in both cells of our sequencing data was set for further study. MiR-30a-5p mimics, inhibitor and their corresponding negative controls were applied in OC cells. Then the cell proliferation, migration, and invasion of different groups were analyzed via cell counting-kit 8 (CCK8), wound healing, and Transwell analyses. Besides, ZBE2 and LDH2 expressions were detected by qRT-PCR.

**Results:**

Combined with the data report of miRNA microarray technology, we set miR-30a-5p as our target miRNA to analyze its molecular function in regulating proliferation, migration, and invasion in OC cells. Our results showed that the miR-30a-5p overexpression could significantly enhance the capability of proliferation, migration, and invasion of HO8910 and HO8910PM cells, whereas the miR-30a-5p inhibition showed the opposite tendency (all P < 0.05). Besides, miR-30a-5p may be involved in these oncogenic processes through the upregulation of ZEB2 and LDH2.

**Conclusion:**

Our results demonstrate that exosome-transmitted miRNA-30a-5p promotes the malignant behavior of OC cells, which may be served as a promising diagnostic and prognostic marker for patients with OC.

## Background

Ovarian cancer (OC) is one of the most life-threatening gynecological malignant tumors in females globally, the 5-year survival rate of which is about 46%, ranking seventh among common cancers in the female population and eighth among cancer-related deaths overall [1, 2]. Due to the non-typical clinical manifestations and the absence of diagnostic biomarkers with good specificity and sensitivity in the early stage, 70–90% of the patients missed the optimal time for treatment once they were diagnosed [3]. As reported by Lancet in 2018, the average 5-year survival rate of OC patients in China remained virtually unchanged for a decade [1]. Therefore, it is crucial to develop reliable biomarkers for early diagnosis of ovarian cancer.

At present, common diagnostic approaches for OC include transvaginal ultrasonography, bimanual rectovaginal pelvic examination, and the serum level of CA125. However, traditional pelvic examination lacks sensitivity and specificity [4]; although the CA125 serum level is elevated in 80% of OC patients, the use of this biomarker for early detection is limited, since only 50% of the patients suffering from OC at Stage I have increased CA125, thus it cannot be regarded as a satisfactory serum biomarker for OC screen [5]. Moreover, the CA125 serum level is also elevated in a certain number of females with benign ovarian lesions as well as in healthy women, limiting its specificity as a diagnostic biomarker [6]. Till now, no highly efficient biomarker has been identified for OC.

Exosomes are extracellular vesicles of 50–150 nm diameter, widely distributed in body fluids and acting as cargo for various molecular content (miRNA, LncRNA, DNA, protein, enzyme) [7]. They are highly stable, which can reflect the origin of the cell [8]. The fusion of exosomes with target cell membranes facilitates the transfer of cell surface molecules and receptors from donor to recipient cells [9]. Burgeoning studies have reported that several types of cancer cells release exosomes at a higher rate compared to normal cells, and contribute significantly in instituting a milieu that supports neoplastic proliferation, angiogenesis, invasion, and metastasis [10–12].

Micro-ribonucleic acid (miRNA), the most abundant long noncoding RNAs transmitted by exosomes, has been revealed to be a potential marker for OC since 2008 [13]. Although a web-based database contains information on the exosome-transmitted-RNA, the detailed relationships between miRNAs, exosomes and OC are still yet to be understood. We have identified the differentially expressed exosomal miRNAs from OC cells by microarray and subsequent bioinformatics analysis, and confirmed that miR-30a-5p is stably expressed in both cell lines. Hence, miR-30a-5p was set as the target miRNA to analyze its molecular function in OC cells. In the present study, we aimed to determine the possible roles of miRNAs derived from exosomes in the early diagnosis of OC through miRNA microarray, additionally, exploring the underlying mechanisms of miRNAs in the OC progression.

## Results

### The isolation and identification of OC cell-derived exosomes

**Fig. 1:**
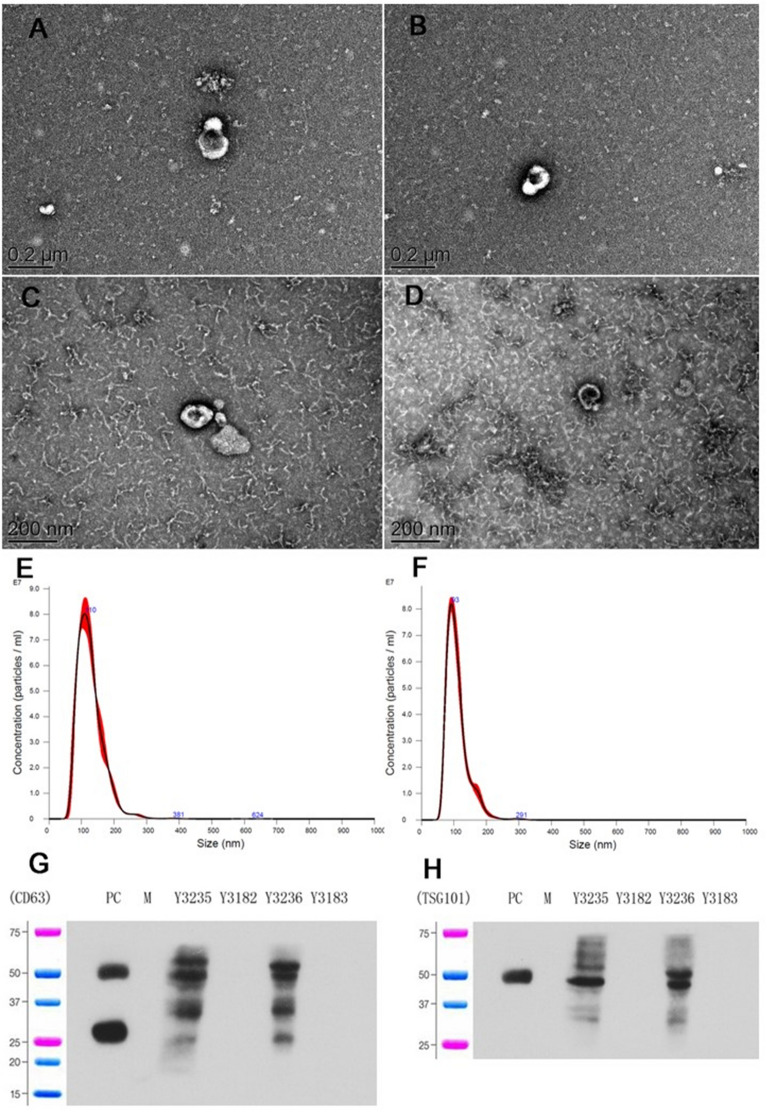
Exosome isolation and identification. **A**–**D** Exosomes were represented as classic cup-shaped vesicles surrounded by two layers of membrane under electron microscopy. **E**, **F** The sizes of isolated exosomes were detected by NanoSight technology. **G**, **H** Common exosomal markers (CD63 and TSG101) were detected by western blot. *PC* positive control, *M* marker

Two OC cell lines were applied in this study, including the highly invasive ovarian cancer cell line HO8910PM and its parent cell line HO8910 for exosome isolation. Cells were cultured in the serum-free and exosome-free medium, and exosomes were isolated from the supernatant using multi-step centrifugation. The features of isolated exosomes were identified by electron microscopy, showing that classic cup-shaped vesicles were surrounded by two layers of membrane (Fig. 1A–D), and their sizes ranged from 30 to 150 nm via NanoSight technology (Fig. 1E, F). Common exosomal markers (CD63 and TSG101) were detected by western blot (Fig. 1G, H). Both results were confirmed the qualified exosome isolation from OC cells.

### Microarray identification of differentially expressed exosomal miRNAs from OC cells

**Fig. 2:**
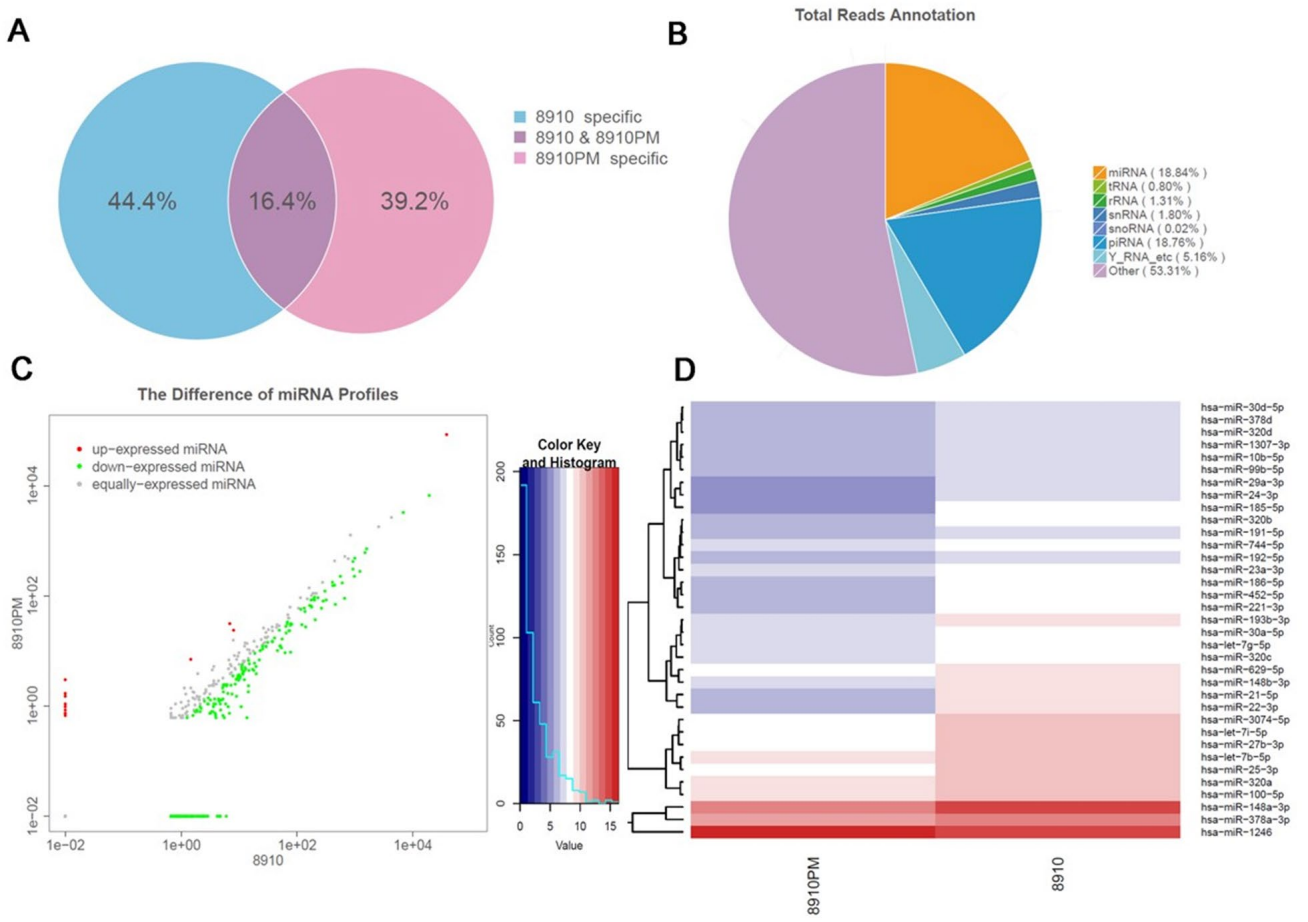
MiRNA microarray information of exosomes derived from OC cells. **A** Common and unique reads between 8910 and 8910PM cells shown by Venn diagram. **B** Total reads annotation of miRNA microarray in isolated exosomes. **C** The scatter diagram of miRNAs expression difference between two OC cell lines. **D** The expression difference of miRNAs shown by cluster analysis

To identify differentially expressed exosomal miRNAs from OC cells, miRNA microarray technology was utilized. The common and unique reads between 8910 vs. 8910PM cells were shown by Venn diagram (Fig. 2A); the filtered reads were compared with the authoritative miRNA database (miRBase version 21, https://www.mirbase.org.) and then annotated the known miRNAs (Fig. 2B). The expression difference of miRNAs between two OC cell lines is represented in Fig. 2C. A total of 2589 miRNAs were determined, among them, 13 miRNAs were overexpressed and 245 miRNAs down-expressed in 8910PM compared with 8910 cell line (P < 0.05), other 2331 miRNAs expressions showed no significant difference in two OC cell lines (consistent tendency). In addition, the results of cluster analysis were represented in Fig. 2D, indicating that miR-30a-5p has a similar and stable expression level in two cell lines (P = 0.63). Combined with the data report of miRNA microarray technology, we set miR-30a-5p as our target miRNA to analyze its molecular function in regulating proliferation, migration, and invasion in OC cells.

### MiR-30a-5p promoted proliferation of ovarian cancer cells

**Fig. 3:**
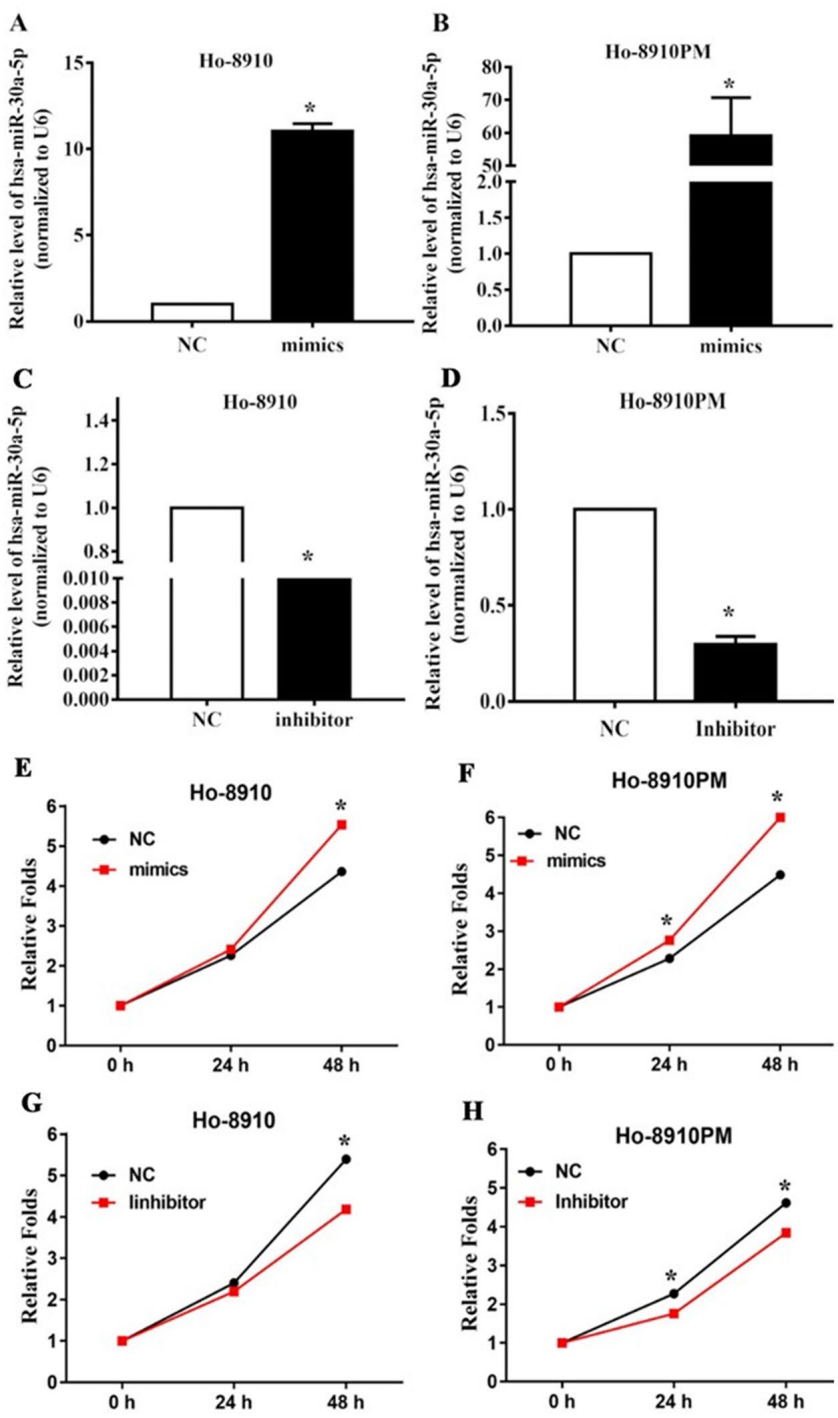
The effect of miR-30a-5p on proliferation in OC cells. **A**–**D** Relative mRNA expressions of miR-30a-5p in 8910 and 8910PM cells transfected with miR-30a-5p mimics, miR-30a-5p inhibitor and their corresponding negative controls (NC) by qRT-PCR, respectively. U6 was used as a loading control. **E**–**H** The proliferation of 8910 and 8910PM cells was measured by CCK-8 assay. *P < 0.05, **P < 0.001

To investigate the biological function of miR-30a-5p in H8910 and H8910 PM cells, miR-30a-5p mimics, miR-30a-5p inhibitor and their corresponding negative controls (NC) were applied to up- or down-regulate the expression level of miR-30a-5p, miR-30a-5p mimics/NC and miR-30a-5p inhibitor/NC sequences were shown in Table 1. The results of PCR showed that miR-30a-5p expression was significantly up- or down-regulated by miR-30a-5p mimics and inhibitor in OC cells, respectively (Fig. 3A–D). To ascertain the effects of miR-30a-5p on the proliferation of OC cells, CCK-8 assay was performed in H8910 and H8910 PM cells transfected by miR-30a-5p mimics and inhibitors. The results represented that compared with the NC cells, miR-30a-5p mimics could enhance the proliferative capacities in both OC cell lines (Fig. 3E, F), whereas, cell proliferation was significantly inhibited in OC cells with silenced miR-30a-5p (all P < 0.05) (Fig. 3G, H).

**Table 1:** Detailed information about sequences and primers

	Sequences (5′–3′)
MiR-30a-5p	F—AAAGTGGACATTTGTAGAGA R—CAGGTACAGACGGATATCTTGC
ZEB2	F—AACGAGTGCGGATTTGTAACCAG R—TTGGCAGTAACAGTTGGGCAAG
LDH2	F—TAAGCTTATTTAAATGGTTCCATCGTACCCTGAATGGAAG R—TTCGCCTTTACGCATGGTGGAAGTGAAGTACGAATGCCG
β-Actin	F—CGACAACGGCTCCGGCATGT R—CTAGGGCGGCCCACGATGGA
U6	R—ATACAGAGAAGATTAGCATGGCCCCTG F—ACACGCAAATTCGTGAAGCGTTCCATATTT

### MiR-30a-5p increases migration and invasion of OC cells

**Fig. 4:**
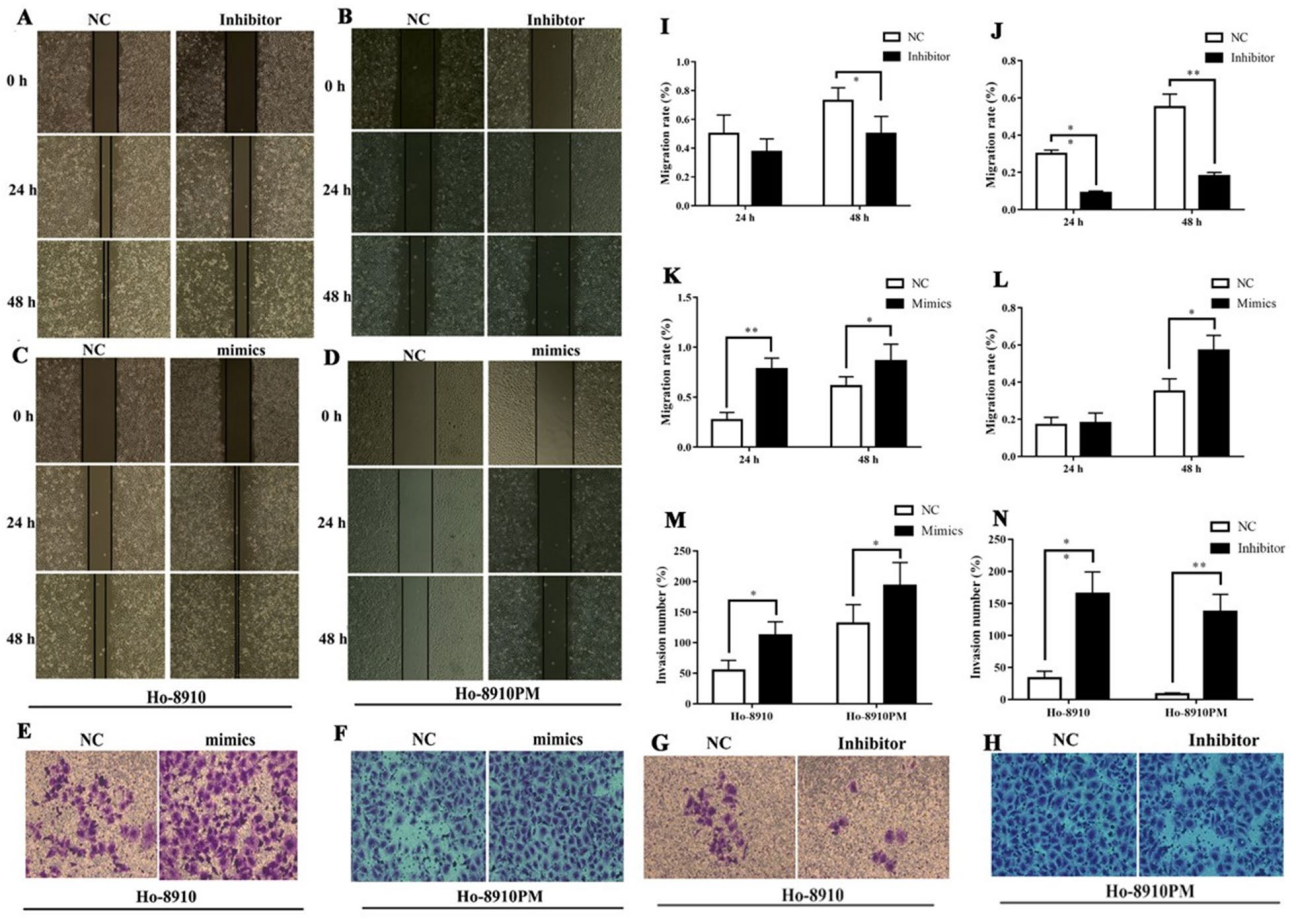
The impact of miR-30a-5p on migration and invasion in OC cells. **A**–**D**, **I**–**L** The migration of two OC cell lines treated with miR-30a-5p mimics or inhibitor was monitored by the wound-healing assay. **E**–**H**, **M**, **N** The invasion of 8910 and 8910PM cells transfected with miR-30a-5p mimics or miR-30a-5p inhibitor detected by Transwell assay. *P < 0.05, **P < 0.001

The migration of OC cells was evaluated using the wound-healing and Transwell assay. Compared with the NC groups, the wound-healing assay showed that the migration of H8910 and H8910PM cells was increased when transfected with miR-30a-5p mimics at 48 h, while attenuated with its inhibitor (all P < 0.05) (Fig. 4A–D, I–L). Consistent with the wound-healing results, downregulation of miR-30a-5p significantly inhibited migration and invasion of HO8910 and HO8910PM cells, as shown in the Transwell assay, however, enhanced miR-30a-5p promoted a significant increase in the migration of OC cells (all P < 0.05) (Fig. 4E–H, M, N). These data together suggested that miR-30a-5p can promote the migration and invasion of OC cells in vitro.

### MiR-30a-5p upregulates the ZEB2 and LDH2 expression in OC cells

**Fig. 5:**
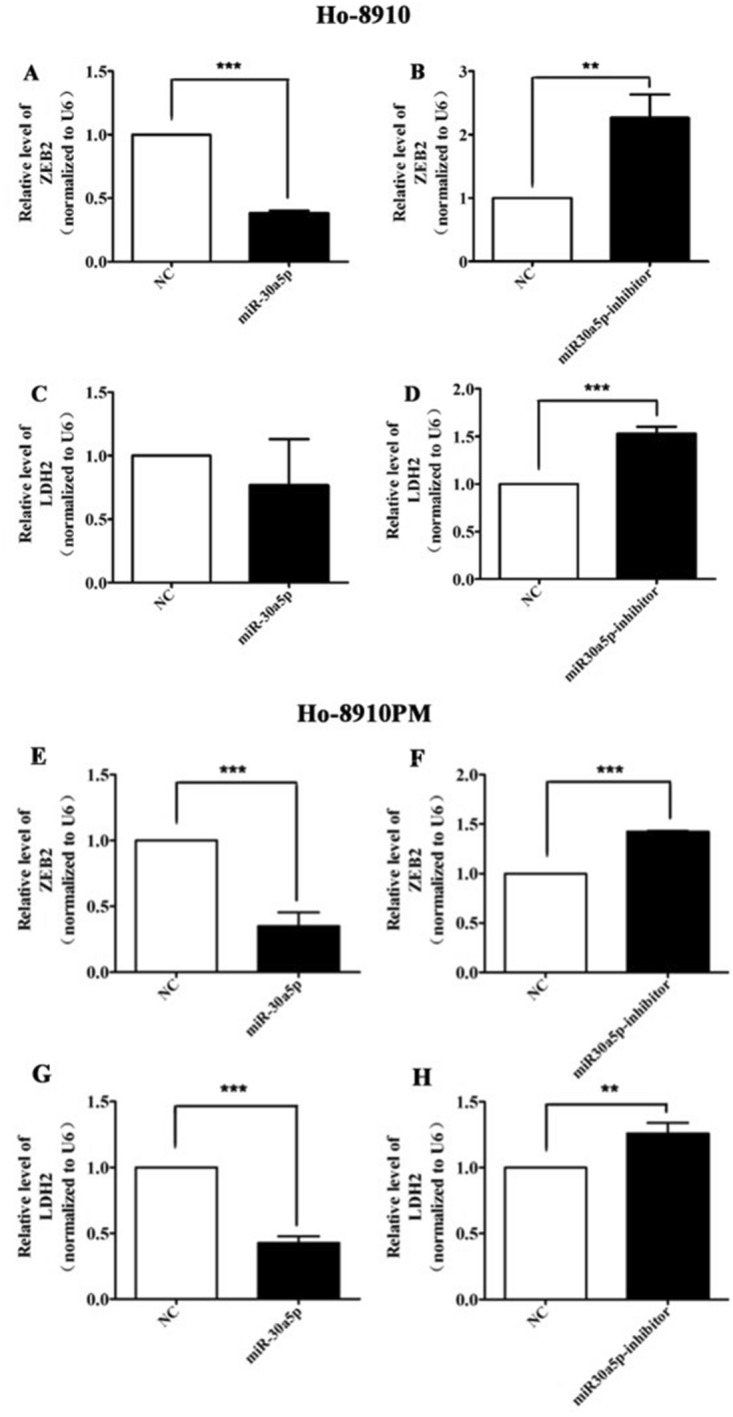
ZEB2 and LDH2 mRNA expressions regulated by miR-30a-5p in OC cells. Relative mRNA expression levels of ZEB2 and LDH2 in 8910 (**A**–**D**) and 8910PM cells (**E**–**H**) transfected with miR-30a-5p mimics or miR-30a-5p inhibitor were examined by qRT-PCR, respectively. β-Actin was used as a loading control. *P < 0.05, **P < 0.001

ZEB2 and LDH2 have been firmly proven that they are crucial oncogenes, playing an important role in OC progression [14–16]. Previous studies reported that ZEB2 and LDH2 expression may be associated with the prognosis of OC patients. In this study, PCR was carried out to detect the expression levels of ZEB2 and LDH2, the results showed that ZEB2 and LDH2 expressions were significantly enhanced in the OC cells transfected with miR-30a-5p mimics, whereas the inhibited in the miR-30a-5p inhibitor group (P < 0.05) (Fig. 5).

## Discussion

Ovarian cancer (OC) is the most frequent cause of death among gynecologic malignancies. Due to its asymptomatic development, most of the patients with OC were in the advanced stage at the time of diagnosis and had a poor prognosis. Despite many years of studies, there is still a lack of reliable diagnostic markers, as well as other diagnostic methods enabling early detection and suitable for screening. Thus, current studies are aimed at finding new biomarkers with diagnostic, prognostic, and predictive potential as well as on the search for new therapeutic targets [17]. In our present study, we identified miR-30a-5p, an abundant exosome-transmitted miRNA from two different OC cell lines with stable expression using Illumina Human HT-12 v4 Expression Bead Chips. After the data analysis of miRNA microarray, miR-30a-5p was regarded as our target miRNA to analyze its molecular function in regulating proliferation, migration, and invasion in OC cells. Interestingly, the miR-30a-5p overexpression could significantly enhance the capability of proliferation, migration, and invasion of HO8910 and HO8910PM cells, whereas the miR-30a-5p inhibition showed the opposite tendency. Besides, miR-30a-5p may be involved in these oncogenic processes through the upregulation of ZEB2 and LDH2.

Since 2008, when the profile of exosomal-transmitted miRNAs used as biomarkers for OC was revealed [13], exosomal-transmitted miRNAs have gradually become a hot spot of OC research. Exosome carries a variety of bioactive substances, playing a key role in intercellular communication in several cancers [18–20]. The protein and microRNA content of exosomes may serve as important prognostic and therapeutic biomarkers for OC [21–23]. Talor et al. [13] demonstrated the highly conserved exosomal-transmitted miRNAs between the tumor origin-exosomal miRNA isolated from serum and OC cell line; besides, the expression profile of exosome-transmitted miRNAs from OC patients and their healthy control was different. This is the first time to introduce the exosome-transmitted miRNAs to the diagnosis of OC. There are plenty of studies that reported the correlation between exosome-transmitted miRNAs and OC progression. A previous study showed the upregulated miR-21 from plasma‐derived exosomes in patients with OC [21]; overexpressed exosomal miR‐200b in the serum of patients is clinically associated with short‐term survival as well as a high‐grade (Stage III and IV) of OC, which is also regarded as a potential biomarker for OC diagnosis [24]. Although these studies focus on the different miRNAs in OC, most of them did not represent a completed exosomal miRNAs profile, which is vital to explicate the whole miRNA types derived from exosomes and provide practical guidance for the clinic.

In this study, our miRNA microarray analyzed 2589 miRNAs, among them, 13 miRNAs were overexpressed and 245 miRNAs down-expressed in 8910PM compared with 8910 cell line, 2331 miRNAs expressions had stable expressions in both OC cell lines (including miR-30a-5p). Zhou et al. [6] indicated that miR-30a‐5p is highly expressed in the urine of patients with OC compared with healthy women, as well as in the exosomes of culture medium with OC cell lines, consistent with our microarray results from OC cells; besides, it is reported that the expression level of 30a‐5p was higher in stage I-II ovarian serous adenocarcinoma samples than that in stage III-IV samples, and higher in well or well-moderately-differentiated ovarian serous adenocarcinoma samples than in poorly-differentiated samples. Similarly, we found the overexpression of exosome-transmitted miR-30a-5p in both OC cells, which were HO8910 and HO8910PM cells. Therefore, our study can infer that the upregulation of miR-30a-5p may be associated with earlier stages or low-invasive features of OC cells in vitro.

MiR-30a-5p has been investigated in several types of cancers in recent years, but the controversy persists. Hence, clarifying the underlying mechanism of miR-30a-5p is urgently required. Liu et al. [25] demonstrated that miR-30a-5p inhibits the proliferation, migration and invasion capability by targeting SOX4 in melanoma cells. While Jia et al. [26] confirmed that miR-30a-5p acts as an oncogenic miRNA in glioma, which could regulate glioma cell growth by targeting SEPT7. In the research of OC, Zhou et al. [6] first found that miR-30a-5p was increased in the urine of OC patients, and miR-30a-5p suppression significantly inhibited the cell proliferation and migration of OC. Based on our miRNA microarray results, we came up with that miR-30a-5p was highly stable in OC-derived exosomes. Notably, the capabilities of proliferation, migration, and invasion were significantly enhanced in both HO8910PM and HO8910 cells transfected with miR-30a-5p mimics, also, miR-30a-5p overexpression could upregulate the protein levels of ZEB2 and LDH2, which were related to the worse tumor classification and poor outcome of OC patients [14–16]. Hence, exosome-transmitted miR-30a-5p acts as an oncogenic miRNA in ovarian cancer, and the tissue-specific expression of miR-30a-5p makes it suitable as a candidate biomarker for OC.

## Conclusion

To the best of our knowledge, this is the first use of microarray technology to investigate the exosome-transmitted miRNA profiling of OC. Altogether, our results demonstrate that exosome-transmitted miRNA-30a-5p promotes the malignant behavior of ovarian cancer cells, which may serve as a promising diagnostic and prognostic marker for patients with OC.

## Methods

### Cell culture

Human ovarian cancer cell lines H8910 and H8910PM were purchased from Shanghai institutes for biological sciences. Both cell lines were routinely tested for mycoplasma, then cultured in a humidified atmosphere of 5% CO_2_ at 37 °C. The H8910 and H8910PM cells were maintained in 90% Dulbecco’s modified Eagle’s medium (DMEM; GIBCO, Life Technologies), with 10% fetal bovine serum (FBS; Thermo Fisher Scientific) and 1% penicillin–streptomycin (Thermo Fisher Scientific).

### Exosome isolation

Cells were cultured with DMEM containing exosome-depleted FBS for 48 h. When 90% confluency was reached, the culture supernatant was collected, and the exosome was obtained by serial centrifugation as previously described [27]. Briefly, the supernatant was centrifuged at 2000×*g* for 20 min to discard debris and dead cells. Then the supernatant was collected and centrifuged at 10,000×*g* for 30 min followed by an additional step of ultracentrifugation at 100,000×*g* for 70 min purified by total exosome isolation reagents (from cells) (Life Technologies), according to the manufacturer’s manual. The cleaned exosomal pellet was finally suspended in 100 µl of PBS and stored at − 80 °C for further use.

### Transmission electron microscopy

Isolated exosomes were washed in PBS, filtered and ultracentrifugation was performed at 200,000×*g* for 1 h to re-pellet the exosomes. The exosome pellet was resuspended and fixed in PBS containing 4% paraformaldehyde, followed by spreading onto glow-discharged formvar-coated copper mesh grids. The sample was stained with 2% uranyl acetate for 5 min, then the grids were photographed under a JEOL 100 CX electron microscope/JEM 1010 transmission electron microscope operated at 80 kV.

### Western blot

The total cell lysate was prepared using RIPA buffer (Beyotime Biotechnology, Shanghai, China) and quantified with the BCA assay (Beyotime Biotechnology). The isolated 50 µg samples were separated by 10% sodium dodecyl sulphate-polyacrylamide gel electrophoresis, and then transferred to the PVDF membrane (Millipore, USA). The membrane was then blocked for 2 h with 5% non-fat milk and probed with primary antibody of CD63 (1:1000, ab193349, Abcam, USA), TSG101 (1:1000, ab83, Abcam) and GAPDH (1:1000, 30202ES60, Yeasen, China) overnight. The secondary antibody was applied for 1 h of incubation at room temperature. The membrane was then immersed in the enhanced chemiluminescence reaction solution (Millipore, USA) in a dark room. Protein band intensity was quantified by densitometry using Image Lab software (Bio-Rad, Hercules, USA) and normalized to the GAPDH level.

### Gene expression analysis of exosomal RNA

Exosomal RNA was extracted from the isolated exosomes using exoRNeasy Midi Kit (50) (Item No. 77144, Qiagen, Germany). Differential gene expression analysis between H8910 and H8910PM was performed using Illumina Human HT-12 v4 Expression Bead Chips. Raw reads set was obtained by TM Illumina HiSeq™ 2500 sequence through the process of removing the joints at both ends, low-quality reads and decontamination. After achieving the clean reads, the distribution of sequence length and the consensus sequence among samples were analyzed. Clean reads were performed as classification and annotation, thus obtaining the composition and expression information of various sRNAs in the sample.

### qRT-PCR analysis

Total cell RNA was extracted using TRIzol Reagent (Invitrogen, USA). cDNA was synthesized by using the Reverse Transcription Kit (Biosystems, Thermo Fisher Scientific, USA) based on the manufacturer’s manual. The primer sequences of miR-30a-5p, ZBE2, LDH2, β-actin and U6 (synthesized by Sangon, Shanghai, China) were listed in Table 1. The quantitative real time-PCR (qRT-PCR) reaction was conducted using ABI SYBR Green Master Mix (Life Technologies, USA) and was performed with the ABI Prism 7500 Sequence Detection System (Life Technologies). PCR reaction was carried out under the following conditions: 95 °C for 5 min; 40 cycles of 95 °C for 10 s; and 60 °C for 30 s. U6 snRNA was used as an endogenous control to normalize miRNA expression, while β-actin was used as the endogenous control to normalize ZBE2 and LDH2. The relative gene expression was calculated by using 2^−ΔΔCt^ method.

### Cell proliferation viability assay

A total of 2 × 10^3^ μm cells per well were plated into 96-well plates, with 200 µl of culture medium and 20 µl of Cell Counting Kit-8 (CCK-8, Beyotime, China) were added into each well. Each experiment group has five parallel wells. CCK-8 is a kind of yellow solution that can be reduced to orange by active cells, whose absorbance is directly proportional to cell number. After 3.5 h of incubation, the OD value of the liquid in each well was measured by a microreader (Bio-Rad 680) at the wavelength of 450 nm. The cell number was proportional to the OD value.

### Transwell assay

Cell invasion and migration were analyzed using Transwell chambers with Matrigel. 5 × 10^5^ cells in 200 µl serum-free medium were seeded on the upper chambers (for cell invasion assay, the chamber was coated with Matrigel, which was 1:6 diluted in the DMEM). The lower chambers were added with 0.6 mL DMEM supplemented with 10% FBS and 1% penicillin-streptomycin. After incubation for 48 h at 37 °C with 5% CO_2_. The cells on the upper surface of the chamber were removed. The cells invading the lower surface were fixed in 4% paraformaldehyde for 30 min, stained with crystal violet for 15 min, and then photographed under the microscope.

### Scratch wound-healing assay

The cells were cultured into 6-well plates at a density of 3 × 10^5^cells/well. Wounds were created by scratching the cell monolayer with a 200-mL pipette tip. To quantify cell migration, the wound width was measured at 0 h, 24 h, 48 and 72 h. The migration distance was normalized to the initial wound width respectively and compared with the control sample. The experiment was repeated three times with similar results.

### Statistical analysis

The data were provided as the mean ± standard error of the mean (SEM). All statistical analyses were performed with Student’s *t*-test (two groups) or one-way ANOVA (three or more groups) using the SPSS software (IBM SPSS statistics 21; SPSS Inc., Chicago, IL). The statistical charts were made by GraphPad Prism 5 software (GraphPad, CA, USA). A P-value < 0.05 was considered statistical significance.

## Data Availability

The data that support the findings of this study are available from the corresponding author upon reasonable request.

## Abbreviations

OC: Ovarian cancer
miRNA: Micro-ribonucleic acid
CCK8: Cell counting-kit 8
